# Pre-exposure prophylaxis uptake among Black/African American men who have sex with other men in Midwestern, United States: a systematic review

**DOI:** 10.3389/fpubh.2025.1510391

**Published:** 2025-03-06

**Authors:** Oluwafemi Adeagbo, Oluwaseun Abdulganiyu Badru, Prince Addo, Amber Hawkins, Monique Janiel Brown, Xiaoming Li, Rima Afifi

**Affiliations:** ^1^Department of Community and Behavioral Health, College of Public Health, The University of Iowa, Iowa City, IA, United States; ^2^Arnold School of Public Health, University of South Carolina, Columbia, SC, United States

**Keywords:** PrEP, barriers, facilitators, African American, Black, MSM, Midwest, United States

## Abstract

**Introduction:**

Black/African American men who have sex with other men (BMSM) are disproportionately affected by HIV, experience significant disparities in HIV incidence, and face significant barriers to accessing HIV treatment and care services, including pre-exposure prophylaxis (PrEP). Despite evidence of individual and structural barriers to PrEP use in the Midwest, no review has synthesized this finding to have a holistic view of PrEP uptake and barriers. This review examines patterns of, barriers to, and facilitators of PrEP uptake among BMSM in the Midwest, United States (US).

**Methods:**

Five databases (CINAHL Plus, PUBMED, PsycINFO, SCOPUS, and Web of Science) were searched in March 2023. We included studies that focused on BMSM in the Midwestern states; only empirical studies (either quantitative or qualitative or both) were considered. We synthesized the qualitative data and teased out some of the factors inhibiting or facilitating PrEP uptake among BMSM.

**Results:**

We screened 850 articles, and only 22 (quantitative: 12; qualitative: 8; mixed methods: 2) met our set eligibility criteria. Most of the studies were conducted in Chicago. Most BMSM use oral than injectable PrEP. Uptake of PrEP ranged from 3.0 to 62.8%, and the majority reported a prevalence of less than 15%. The barriers include PrEP awareness, PrEP access, PrEP stigma, side effects, PrEP preference, socioeconomic status, medical insurance and support, partner trust, trust in the health system, and precautions with sexual partners. The identified PrEP facilitators include PrEP use until HIV is eradicated, friend influence, experience with dating men living with HIV, safety, phobia for HIV, disdain for condoms, and power to make decisions.

**Conclusion:**

Our review summarized patterns of, barriers to, and facilitators of PrEP uptake among BMSM in the Midwest, United States. The low PrEP uptake of BMSM was primarily attributed to mistrust in the health system and low socioeconomic status. Multimodal and multilevel strategies are needed to improve PrEP uptake among BMSM, including improving the marketing of PrEP to BMSM and removing financial barriers to accessing PrEP service.

## 1 Introduction

Approximately 12% of the United States (US) population were Non-Hispanic Black/African American in 2019 (1), and yet 37.4% of people living with HIV (PLWH) are non-Hispanic Black/African Americans (2). Similarly, Black/African American men who have sex with other men (BMSM) experience significant disparities in HIV incidence, access to HIV care, and prevention across all age groups (3–5). Of the estimated 37,981 new HIV diagnoses in the US in 2022, 70% were among men who have sex with other men (MSM), including BMSM (34%) (6). The Centers for Disease Control and Prevention (CDC) estimated that one in two BMSM will be diagnosed with HIV in their lifetime (7), and BMSM are eight times more likely to be diagnosed with HIV than White MSM in their lifetimes (8).

As of 2022, in the Midwest US, males (79.3%) and Blacks (41.6%) were more likely to be living with HIV, and male-to-male sexual contact (79.2%) was the commonest mode of transmission. Also, eight in ten (81.6%) of new HIV cases were among MSM (9). Furthermore, BMSM are more burdened with HIV than non-Hispanic Whites. For example, Mustanki and colleagues, in their cohort study, found that HIV is more common among BMSM than their Hispanic and non-Hispanic White counterparts (10). Similarly, BMSM are 10 times more likely to be living with HIV than non-Hispanic Whites in Iowa (11). Lack of medical insurance for HIV preventive care, historical discrimination, and structural racism, such as institutional racism and homophobia, are some of the root causes of HIV disparities in the US (12, 13).

Recently, the US government published a plan for “Ending the HIV Epidemic”(EHE) by 2030 (14–16). One of the four strategic goals of the EHE initiative is wider pre-exposure prophylaxis (PrEP) coverage, especially among populations at higher risk of HIV acquisition (14). PrEP is a biomedical medication that, when used consistently by an HIV-negative person, reduces the chances of contracting HIV for all populations, including priority populations such as those who inject drugs and BMSM (12, 17). Effective use of PrEP by BMSM will reduce the HIV burden by halting ongoing HIV transmission and contributing to MSM community-level protection (18). Several studies (including clinical trials) have found PrEP highly effective for HIV prevention, particularly for those at risk, including BMSM (19–21). Despite the documented benefits of PrEP as an effective HIV prevention method, the uptake of this biomedical medication to prevent HIV is very low across the US, including among BMSM (3, 22). According to recent estimates from the CDC, only 30% of the 1.2 million people in the US who might benefit from PrEP were prescribed PrEP in 2021 (23). Common barriers to PrEP uptake included factors such as low socioeconomic status, health insurance, medical mistrust, misinformation about PrEP, as well as racism, discrimination, healthcare providers’ negative attitudes, and PrEP access (12, 22, 24, 25). The COVID-19 pandemic further exacerbated PrEP uptake by intensifying several of these barriers (26, 27).

Several PrEP-related reviews in the US have focused on PrEP uptake, barriers, and facilitators among only MSM or the general population, including MSM and transgender persons (28–30). To our knowledge, no review has synthesized the uptake, barriers, and facilitators of PrEP use among BMSM in the US Midwest states despite increasing rates of new HIV diagnoses cases and rates since 2020 in the Midwest (31), and that 3 of the 7 States with the greatest unmet need for PrEP among Black people were in the Midwest (32). This systematic review aims to investigate patterns of PrEP uptake and identify barriers and facilitators to PrEP uptake among BMSM in the Midwest, US. This would inform the development of interventions to increase PrEP uptake among this priority population and help achieve the goals of the EHE initiative in the US.

## 2 Methods

This systematic review was conducted in line with the Updated Preferred Reporting Items for Systematic Review and Meta-Analysis Protocols (PRISMA-P) guidelines (33).

### 2.1 Eligibility criteria

Our eligibility criteria followed the population/participants, interventions, comparison, outcome, and study design (PICOS) framework (34). We included empirical studies conducted solely or partly among BMSM in the Midwestern states of the United States. Commentaries, letters to the editor, or expert opinions were not considered. We focused on studies with a primary or secondary focus on PrEP uptake, barriers, or facilitators. We did not limit our search by language or time.

### 2.2 Search strategy

CINAHL Plus, PUBMED, PsycINFO, SCOPUS, and Web of Science were searched in March 2023 using relevant keywords (such as PrEP, barriers, and specific Midwest states), Medical Subject Headings (MeSH) terms, and Boolean operators. For example, we search PubMed with the following terms: (((Barrier* OR problem* OR reluctan* OR concern* OR stigma OR perception OR belief OR attitude OR enabler* OR Motivator* OR facilitator* OR encouragement OR predictor* OR determinant* OR engagement OR uptake OR initiation OR Use OR Utilization OR Utilization OR Compliance OR Adherence) AND (“Pre-Exposure Prophylaxis”[Mesh] OR “pre-exposure prophylaxis” OR PrEP OR Truvada OR Descovy)) AND (“men who have sex with men” OR MSM OR Gay* OR “male couple*” OR homosexual* OR “transgender wom*” OR “trans wom*” OR “bisexual men”)) AND (Iowa OR “Midwest region” OR “Midwestern region” OR “Midwest state*” OR Illinois OR Indiana OR Kansas OR Michigan OR Minnesota OR Missouri OR Nebraska OR “North Dakota” OR Ohio OR “South Dakota” OR Wisconsin). We did not limit our search by date or language. The comprehensive search strategy for other databases is in the Supplementary material. Rayyan, an online article manager (35), was used for the article screening process. Two reviewers (OAB and PA) met to finalize the study eligibility criteria before screening. One reviewer (OAB) removed duplicate articles; two reviewers (OAB and PA) independently performed title and abstract screening. Both reviewers resolved all disagreements through discussions. References of all articles that met our eligibility criteria were manually searched for additional relevant articles.

### 2.3 Data extraction procedure

We extracted the following details from each article that met the set eligibility criteria: author and year of publication, Midwest state, study design, sample size and technique, type of PrEP (pills or injectables), analysis type, BMSM sociodemographic information (i.e., race and age), PrEP uptake, barriers to and facilitators of PrEP use. One reviewer (OAB) extracted all the details, while another reviewer (OAA) checked for accuracy.

All the articles that met the eligibility criteria were subject to methodological rigor assessment using the appropriate Joanna Briggs Institute tools (depending on the study design); one reviewer (OAB) performed this process, which was verified by another reviewer (OAA).

### 2.4 Data analysis

The quantitative findings were summarized descriptively. We synthesized the qualitative data and identified the common factors inhibiting or facilitating PrEP uptake among Black MSM.

## 3 Results

**Figure 1:**
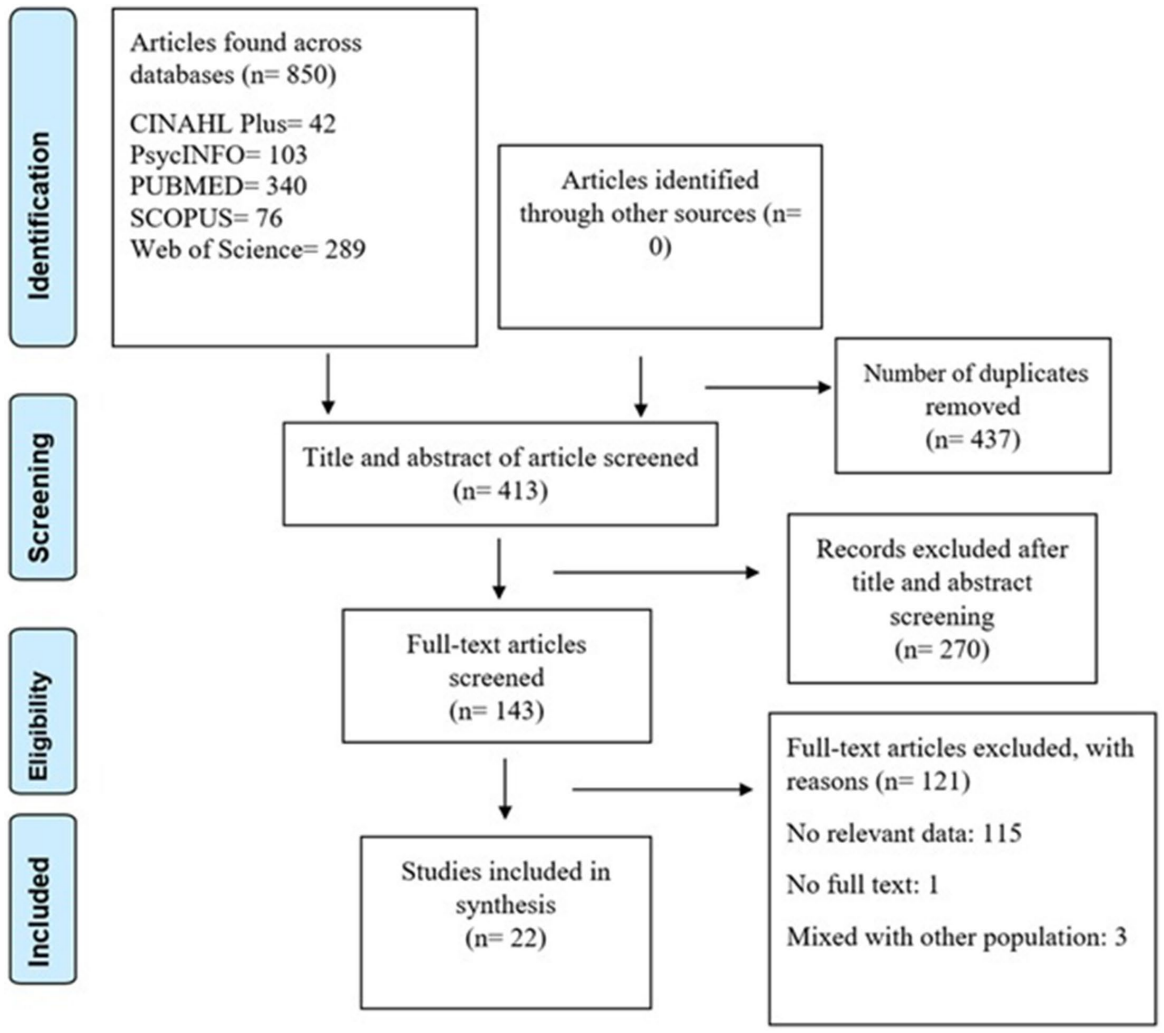
Search strategy flowchart.

The systematic search across five databases produced 850 articles. The duplicates were 437, leaving 413 articles for title and abstract screening. Of these, 270 were excluded as they did not meet our eligibility criteria; the remaining 143 articles were subject to full-text screening. A further 121 articles were excluded for lack of relevant information and having no distinct information for BMSM. Therefore, only 22 articles that met the eligibility criteria were included in this review (Figure 1). All the studies were judged to have high methodological rigor (Table 1).

**Table 1:** Characteristics of the included studies.

Author	State	Study design	Sample size	Study sampling	PrEP type	Data analysis	Participant’s characteristics	PrEP uptake	Evidence level
Biello et al. (36)	Chicago	Qualitative: FGD	38; 8 from Chicago Black: 69.4%	Purposive	Injectable	Content coding	YBMSM 15–29 years	-	High
Downing et al. (47)	Detroit in Michigan (other states: Atlanta and New York)	Qualitative: IDI	26; 8 from Chicago	Consecutive (Banner advertisements placed on social media websites such as Facebook)	Pill	Content analysis	GBMSM 19 to 62 years Mean age: 29	-	High
Hall et al. (37)	Chicago	Qualitative: IDI from RADAR cohort From mixed methods study	28	NA	Pill	Thematic	PrEP-using MSM Mean age: 25.57 ± 1.93	-	High
Kelly et al. (49)	Wisconsin	Cohort	33	Respondent Driven Sampling (Seed)	Pill	Inferential	BMSM Average age: 27 years	Baseline: 1 (3.0%) 3 months follow-up: 4 (12.1%)	High
Khanna et al. (39)	Chicago	Cohort (uConnect)	266	Respondent Driven Sampling	Pill	Descriptive	YBMSM 16–29 years	Baseline: 10 (3.8%) Wave 2: 16 (6.0%)	High
Khanna et al. (38)	Chicago	Cohort (uConnect; Baseline)	622	Respondent Driven Sampling	Pill	Inferential	YBMSM 16–29 years	3.6%	High
Lancki et al. (40)	Chicago	Cohort (uConnect)	618 289 for PrEP response	Respondent Driven Sampling	Pill	Inferential	BMSM 22.1 ± 0.3	Wave 1 (Baseline): 4% Wave 2: 6.6% Wave 3: 10.1% Wave 1–3: 42 (14.5%)	High
Morgan et al. (41)	Chicago	Baseline: Cross-sectional from Cohort (RADAR)	885 Black: 259 (29.3%)	Snowball, including social media and venue-based	Pill	Inferential	BMSM 16–20 years 20.8 ± 2.8	19 (7.3%)	High
Mustanki et al. (10)	Chicago	Baseline: Cross-sectional from Cohort (RADAR)	1,015 Black: 344 (33.9%)	Diverse methods: Snow ball, etc.	Pill	Inferential	BMSM 16–29 years	7.14% past 6 months	High
Patel et al. (48)	Missouri	Qualitative: IDI	26 BMSM	Snowball	Pill and injection	Inductive	BMSM Median age: 27 (24–30)	-	High
Phillips et al. (42)	Chicago	Cross-sectional from Cohort (RADAR)	906 MSM: 257	Respondent Driven Sampling	Pill	Inferential	YMSM Median age: 20.2	20 (7.8%)	High
Quinn et al. (50)	Wisconsin (Milwaukee)	Qualitative: FGD	44 BMSM	Convenience	Pill	Inductive	BMSM Mean age: 22 ± 2.3; range 18–25	-	High
Quinn et al. (52)	Milwaukee Minneapolis Detroit Kansas	Qualitative: 6 FGD	36 BMSM	Purposive	Pill	Inductive	YBMSM 25.9 ± 3.6 Range 20–30 years	Current use: 27 (75%) Previous use: 9 (25%)	High
Quinn et al. (53)	Milwaukee Minneapolis Detroit Kansas	Qualitative: 4 FGD	44 BMSM	Purposive	Pill	Inductive	YBMSM 22.3 ± 2.3	Current: 8 (18%) Previous use: 2 (5%) Never: 34 (77%)	High
Quinn et al. (51)	Cleveland Milwaukee	Qualitative: IDI	46 BMSM	Purposive	Pill	Thematic content (Inductive)	BMSM 25.2 ± 3.8	Current: 9 (20%) Previous use: 2 (4%)	High
Quinn et al. (8)	Cleveland Milwaukee	Mixed-method (quantitative)	283 YBMSM	Purposive	Pill	Inferential	YBMSM 21.70 ± 2.75 Range 16–25 years	Current: 37 (13%) Previous use: 23 (8%)	High
Remy et al. (4)	Missouri	Qualitative: IDI	12 BMSM	Purposive Convenience Snowball	Pill	Inductive	BMSM Modal age group: 26–34 (66.7%)	-	High
Schneider et al. (43)	Chicago	PrEPChicago intervention (baseline)	423 YBMSM Intervention: 209 Control: 214	Respondent-driven	Pill	Inferential	YBMSM Mean age intervention group: 26.1 ± 4.2 Control group: 25.7 ± 4.3	Intervention group: 20 (9.6%) Control group: 20 (9.4%) Total use: 9.5%	High
Schueler et al. (44)	Chicago	Cross-sectional	218 Black 190 (88.8%)	Snowball	Pill	Inferential	BMSM 29.8 ± 10.4	11 (5.1%)	High
Schuyler et al. (24)	Chicago	Cross-sectional with open-ended questions	160	Quota	Pill	Inferential and content analysis	AAYMSM 17–24 years	22 (13.8%)	High
Timmins et al. (45)	Chicago	Cross-sectional (N2 Cohort Baseline)	173	Snowball	Pill	Inferential	BMSM 25.2 ± 3.9	56 (32.4%)	High
Young et al. (46)	Chicago	Cross-sectional (Baseline) (PrEP Chicago)	423 Intervention: 209 Control: 214 Uptake: 406	Respondent-driven	Pill	Inferential	BMSM Mean: 26	40 (9.9%)	High

FGD, focus group discussion; IDI: In-depth interview; YBMBM, Young Black men who have sex with men; AAYMSM, African American young men who have sex with men; GBMSM, Gay, bisexual, and other men who have sex with men.

### 3.1 Study design and data collection methods

Of the 22 studies, 15 (62.5%) were conducted in Chicago (10, 24, 36–46). The remaining studies were conducted in Michigan (47), Missouri (4, 48), and Wisconsin (8, 49–51); two studies focused on four regions: Detroit, Kansas, Milwaukee, and Minneapolis (52, 53).

Twelve (59%) studies were quantitative (10, 24, 38–46, 49), and eight (33%) were strictly qualitative (4, 36, 47, 48, 50–53). Two (8%) studies adopted a mixed methods design (8, 37) but reported either quantitative findings only (8) or qualitative findings only as part of a broader study (37). It is important to stress that seven of the cross-sectional studies analyzed a portion of results from a cohort study (10, 41, 42, 44–46). For studies that conducted qualitative designs, whether qualitative only or from a mixed methods study, five used an in-depth interview approach (4, 37, 47, 48, 51), while four used focus group discussions (36, 50, 52, 53).

### 3.2 Pattern and uptake of PrEP

Most of the studies (*n* = 22, 92%) focused only on oral PrEP (i.e., pills), while Biello et al. (36) focused on injectable PrEP and Patel et al. (48) focused on both pills and injectable PrEP. Uptake of PrEP differed across the 12 studies that quantified PrEP uptake, ranging from 3.0 to 32.4% (8, 10, 24, 38, 40–46, 49). However, overall, the findings reveal that most BMSM may not be using PrEP. For instance, 11 studies (79%) reported a PrEP uptake of less than 15% (10, 24, 38–44, 46, 49); and Timmins et al. (45) reported 32.4%. The two studies that reported baseline and follow-up PrEP use recorded little difference between both periods (39, 49).

### 3.3 PrEP uptake barriers

The barriers to PrEP uptake among BMSM in the Midwest are based on the findings of the included qualitative studies. Several barriers to PrEP uptake emerged, including PrEP unawareness, access, stigma, PrEP side effects, low socioeconomic status, trust in partners, distrust in the health system, and concerns over PrEP adherence (Table 2).

**Table 2:** Barriers and facilitators to PrEP uptake.

Theme	Representative quote
PrEP barriers	
PrEP unawareness	“I would probably go with the condom. I mean, just because I do not really know much about the pills because I never used it before. None of my friends have ever told me they used it. So, I just really have no education on the pill.” (pp. 10) (51)
Side effects	“For starters, I would say take the pill so you can learn about the side effects. Then you can stop at any time. Once you are comfortable with the pill, you could maybe switch over to the injection.” (p. 5) (36)
Low socioeconomic status (SES)	“I do not wanna say resources, because, like, everybody has the same, you know, seem like everybody got the same resources. I was thinkin’ like resources, you know, ‘cuz like you said, you know, the Black community, we gotta lot of stuff on our plate. And not to say, you know, Whites do not have a lot on their plate either but, you know, we are dealing with unemployment, finding jobs, you know, the hood. All the extra stuff, stuff. A lot of stuff that’s on our plate, and so we not really carin’ about PrEP, or whatever…” (p. 5) (50)
Lack of access to PrEP	“access [to PrEP] is a pain in the a**” (p. 7) (4)
Concern over PrEP adherence	“I do not want the pills every day—I would definitely miss some.” (p. 4) Biello et al. (36)
Distrust in the health system	“You would think that some of the younger [Black men], maybe you would not know about Tuskegee experiments or would know about Henrietta Lacks, but you know what? …. they did.” (p. 11) (4)
PrEP stigma	“I just did not feel good carrying it [PrEP] around. So, I stopped. Because it made it look like something it wasn’t, the medication box…So I just stopped, because it looked different. You know, maybe people would think like I was (HIV positive).” (p. 9) (24)
Trust in partner	“I mean, that’s the reason why. My risk is not that high anymore. So, if I felt like if somebody gained that trust and I mean you do not necessarily have that risk, why are you wasting the resources? I mean not to say that you should not still protect yourself, but I just felt like if a person has that trust, you do not necessarily have to worry about it.” (p. 4) (53)
Precaution with sexual partners	“I got PrEP from my doctor and I was taking it. And then I was doing research on it and stopped taking it. Because I was like…do I want to have sex with someone who is HIV positive even if there’s a chance that I will not get it? I was like no, I do not think so…old fashion way.” (p. 8) (24)
PrEP facilitators	
PrEP use until HIV is eradicated	“It’s scary though how it was like you might not have to take it for the rest of your life because sexuality is fluid. So that’s like saying you know I’m settling down with this one partner, he’s negative, I’m negative we can go raw! And then so I do not have to take the PrEP and then I have to take it later, but I do not know if I want to keep. If sexuality is fluid, then it would be best to just stay on PrEP. Anybody having sex should be on PrEP until we have eradicated this disease.” (p. 8) (47)
Friend influence	“I kept telling my friend because he has to take pills every day. I was like, ‘I do not know how you do this. I cannot.’ He was like, ‘You need to go to Walgreens and buy the pill thing for every day.’ I was like, ‘Oh, okay.’ Now, when I did that, I took over the world. I was consistent. I was good. So, that helped.” (p. 7) (37)
Experience with dating men living with HIV	“I started taking PrEP because I dated men who were positive in the past, and so just like he said, another layer of protection. And just arming myself with like the knowledge and doing the independent research and, you know, not stigmatize anybody just because of that, yeah.” (p. 8) (52)
Safety	“I would take the pill most likely every night before I go to bed. I want to be safer taking it every day than whenever I have sex.” (p. 7) (37)
Phobia for HIV	“Just a phobia about catching HIV really. You can be in a monogamous relationship, does not mean your partner’s going to be monogamous. I mean you always have to protect yourself… You got to put the responsibility in your hands.” (p. 8) (52)
Disdain for condom	“I personally do not like wearing condoms. And that’s just because I’m usually the top and I do not like the way condoms feel. So that was a big reason I got on PrEP in the first place, because I found condoms to be very frustrating experience and so I feel more sexier when I do not have to wear a condom when I’m topping. So that’s like my reason for taking PrEP.” (p. 7) (52)
Power and personal autonomy	“It’s the truth, yeah! It gives you the autonomy to really make [sexual] decisions for yourself. So, like whatever fits you sexually. And like then I can turn the conversations to HIV a little bit more confidently, irrespective of what they will or will not share with me.” (p. 8) (52)

Regarding the PrEP barriers, being unaware of PrEP was common among BMSM in the Midwest. Some BMSM reported that they had never heard of PrEP (51). PrEP side effects were inhibiting factors for those who were aware of PrEP (36). Additionally, we found that low socioeconomic status often prevents access to PrEP. Specifically, some BMSM were uninsured compared to their White counterparts and, as a consequence, do not have money to pay for PrEP-related care when needed, exacerbated by the difficulty in securing jobs (50).

Furthermore, BMSM reported structural-related issues in accessing PrEP. For example, many BMSM reported they could not access PrEP in health facilities for reasons such as health workers not being aware of it or refusing to make it available to them, and even when available, the wait time can be very long (4). Another barrier is distrust of the health system. BMSM continues to refer to historical unethical practices experienced by the Black community, such as the Henrietta Lacks and the Tuskegee experiments, as a reason for not using PrEP (4).

BMSM reported that the fear of others knowing that they are using PrEP is a primary reason they are not interested in PrEP uptake (24, 36). According to some of them, they risk being seen with PrEP if they opt to take it and fear that people will assume they are living with HIV (24, 36). There is evidence that the concern of being labeled “HIV positive” led to the discontinuation of PrEP among some BMSM (24). Interestingly, some BMSM in monogamous relationships trust their partner, and they seem not to be interested in PrEP because they perceive their HIV risk to be low, and they do not want to lose their partner’s trust (24, 53). Moreover, some BMSM did not see a need to be on PrEP because they do not intend to have sexual intercourse with an infected person (24).

### 3.4 PrEP uptake facilitators

We found several factors that made BMSM utilize PrEP, including the safety it provides, having friends who use PrEP, fear of HIV, dislike for condoms, and experience dating men living with HIV. Specifically, some BMSM reported that they adhered to PrEP because they had friends who were on PrEP (37). Furthermore, BMSM were more likely to use PrEP if they had had an experience dating men living with HIV (52), mainly because it protects or provides safety against HIV infection (37, 53).

Another interesting reason for PrEP uptake was the sexual autonomy and power it provides because BMSM on PrEP have more flexibility in sexual decision-making (52). Moreover, they felt that it was better to be on PrEP than use condoms, which they perceived to be “frustrating” and less enjoyable (52). BMSM also alluded that they choose to use PrEP because of fear of contracting HIV (52), and many chose to remain on PrEP until HIV is “eradicated” (47).

## 4 Discussion

This systematic review assessed barriers and facilitators to PrEP use among BMSM in the Midwestern states. The uptake of PrEP appears low among BMSM in the Midwest. Most of the studies that quantified PrEP uptake reported a prevalence of less than 15%, and we found several barriers that could influence the lack of PrEP use among BMSM from qualitative studies only. This leaves a gap that needs to be filled by researchers interested in PrEP-related research.

### 4.1 PrEP uptake barriers

One major barrier to PrEP uptake was the lack of PrEP awareness and knowledge among BMSM. Coukan et al. (54) found a similar issue in their review of barriers to PrEP among underserved populations and MSM in the United Kingdom (UK). Knowledge and awareness of PrEP should precede its access and uptake. This calls for more sensitization of PrEP not just for BMSM in the Midwest but also for all key or priority populations globally. Lack of awareness was not limited to BMSM alone; we also found evidence of a lack of knowledge and awareness of PrEP among healthcare providers. Previous US reviews have also reported a similar finding from a pool of studies across several states (30, 55). Lancki et al. (40) reported that the extent to which healthcare providers influence low PrEP awareness and uptake among those who need it might be uncertain, but it is worrisome as it directly impacts counseling and PrEP prescription for BMSM in the US, and could have a negative effect on interventions (30, 56).

Interventions to improve PrEP awareness should be bidirectional, focusing on BMSM (and other priority populations) and healthcare providers; the latter may be more important than the former. An earlier systematic review of healthcare providers’ barriers to PrEP in the US found no intervention tailored toward the improvement of healthcare providers’ knowledge (55); Pleuhs and colleagues also reported the willingness of healthcare providers to prescribe PrEP after an educational intervention; this gap needs urgent attention (55).

Another barrier that impedes on BMSM’s PrEP uptake was low SES. We found that BMSM with low SES were less likely to initiate PrEP and other health services. This perhaps led to complete neglect of the healthcare system and interest in PrEP. Some BMSM prioritized earning a living in the face of unemployment, which is perceived to be more prevalent among Black/African American populations (57).

Furthermore, lack of access to PrEP was a major a barrier. BMSM appeared to have issues accessing health facilities to obtain PrEP, similar to the findings of earlier reviews (30, 54). The lack of access to PrEP may be due to financial difficulties, lack of medical insurance, and limited deliveries of PrEP to Black communities (30). Also, despite the implementation of TelePrEP in Iowa to address access and other barriers, only a few African Americans (17/167) initiated PrEP (58). Innovative strategies are required to overcome PrEP access, especially for BMSM and other priority populations (59).

HIV-related stigma was another barrier identified in this review. HIV-related stigma may have a negative effect on PrEP uptake because there are concerns that being on PrEP may attracts social stigma, particularly enacted stigma, and some BMSM were worried about being perceived to be sexually deviant or promiscuous. Other reviews focused on MSM and transgender persons in the US and UK have established a similar observation (28, 30, 54, 55). Public health experts in the HIV space need to sensitize the larger community about PrEP benefits to reduce the PrEP-related stigma.

Moreover, another important barrier to PrEP uptake among BMSM was their knowledge of previous unethical experiments like the Tuskegee experiment with the Black or African American community Throughout history, the health system has not earned the trust of the Black community following previous unethical experiments (e.g., the Tuskegee experiment), and HIV treatment is a famous example, which unfortunately seems to be playing out with PrEP. Lack of trust in the health system was reported by several PrEP-related reviews (28, 30, 54, 55).

### 4.2 PrEP uptake facilitators

Regarding PrEP facilitators, this review found that BMSM were not interested in using PrEP if they trusted that their partners were faithful to them and if they were in a monogamous relationship. We did not find an earlier review with a similar observation among BMSM. Naturally, trust strengthens the bond between couples. Some may argue that having trust in a partner may not be a barrier to PrEP use as there may not be a need to be on PrEP if there are no risks. Interestingly, we also found that trust did not matter to some BMSM as they were on PrEP to limit their risk of contracting HIV. Also, being in a monogamous relationship does not necessarily mean that one’s partner may not have other sexual partners, which led to regular PrEP use by some BMSM. This result corroborates other primary studies conducted in the US and Vietnam (60, 61).

The present review provides some insights into factors that influence PrEP use among BMSM. BMSM were more likely to use PrEP if they had friends living with HIV, perhaps due to perceived susceptibility to HIV. Not surprisingly, those who have previously dated men living with HIV were motivated to be on PrEP to reduce their chances of contracting HIV (62).

This review found that some BMSM prefer condomless sexual intercourse, claiming that condoms reduce pleasure, and choose to reduce HIV risk by being on PrEP. The debate on the sensitivity of condoms has been long discussed. However, it may be acceptable to choose from the range of HIV preventive strategies available since all the preventive strategies have a similar objective – preventing HIV. Also, some BMSM chose to be on PrEP to minimize HIV risk, irrespective of the level of trust for their partners (63).

This review is not without limitations. This is the first review to report about BMSM in the US Midwest. However, the findings may not generalize to other BMSM outside the Midwest or the US because the context may differ. Our coding of the themes may not have captured the true picture of what individual studies intended, as we cannot access the full transcripts and interview guides. Also, a few studies had a small number of non-BMSM participants in their sample, which may have impacted our findings. Moreover, some studies were conducted by the same authors who may have published these studies using the same data and population, which may influence our conclusions. Additionally, many of the previous reviews that we compare our results to focus mostly on urban areas. Therefore, we are not able to differentiate between urban and rural communities in our paper. Also, we could not perform a meta-analysis for quantitative data due to the heterogeneity of the data obtained from the included studies. For instance, some studies had a few participants who were bisexual (men who have sex with men and women, and others had transgender women who have sex with men). A pooled estimate of the uptakes may be possible with a sub-group meta-analysis; however, because of the variation in population and study designs, we may be committing type 2 error due to fewer studies in each subgroup analysis. It was not advisable to pool estimates with traces of heterogeneity and bias (64).

## 5 Conclusion

We synthesized common and ubiquitous barriers to PrEP uptake among BMSM in the Midwestern states of the US, including lack of PrEP awareness by BMSM and healthcare providers, PrEP access, PrEP stigma, and distrust in the health system. For BMSM using PrEP, friends influences, experiences dating other men living with HIV, displeasure from condoms, and fear of HIV were some of the reasons they opted for PrEP. Although these barriers and facilitators are not unique to the Midwest, they are important to consider in developing HIV prevention interventions in the region. Fundamental issues must be addressed to flatten the HIV curve for BMSM and other sexual minority populations. Multimodal and multilevel strategies are needed to improve PrEP uptake among BMSM. Poor knowledge or lack of PrEP awareness is arguably the major barrier to PrEP uptake because intention to act (use PrEP) may be influenced by knowledge of PrEP. Although knowledge of PrEP does not necessarily increase PrEP use (as the history of institutional and structural racism may impede PrEP uptake), PrEP awareness can serve as the entry point to its use. Therefore, the increase in PrEP awareness and knowledge is important. Furthermore, the initiative and funds directed toward HIV treatment can be replicated in HIV prevention, particularly the availability and accessibility of low-cost or free PrEP for users. Moreover, perhaps due to previous unethical experiments and individual experience engaging with HIV care, there is a need to build the trust of those with default mistrust for biomedical innovations through genuine information about PrEP (including its potential side-effects) and community engagements, with an emphasis on Black communities. Biomedical and HIV researchers must show transparency in trials and clinical research to boost the confidence of the target population, such as MSM and other priority populations. Finally, more advocacy on the importance of PrEP and the need to support persons interested in PrEP is needed while discouraging PrEP-related stigma. Innovations like long-lasting PrEP, such as injectable PrEP, and portraying PrEP as a medication for all rather than a specific population with a risk for HIV may help improve the visibility and acceptance of PrEP.

## Data Availability

The original contributions presented in the study are included in the article/Supplementary material, further inquiries can be directed to the corresponding author.
