# Supplementary material for: Pre-exposure prophylaxis uptake among Black/African American men who have sex with other men in Midwestern, United States: a systematic review
Source: Front Public Health. 2025 Mar 6;13:1510391. doi: 10.3389/fpubh.2025.1510391 (PMC11923624; doi:10.3389/fpubh.2025.1510391)
Supplement: Supplementary file 1 [file Table_1.docx]

**SEARCH STRATEGY**

**Table S1: Search strategy for PubMed**

| **SEARCH** | **ALL TERMS** | **HIT** |
| --- | --- | --- |
| **#1** | **Barrier* OR problem* OR reluctan* OR concern* OR stigma OR perception OR belief OR attitude OR enabler* OR Motivator* OR facilitator* OR encouragement OR predictor* OR determinant* OR engagement OR uptake OR initiation OR Use OR Utilisation OR Utilization OR Compliance OR Adherence** | [12,145,449](https://pubmed.ncbi.nlm.nih.gov/?term=Barrier%2A+OR+problem%2A+OR+reluctan%2A+OR+concern%2A+OR+stigma+OR+perception+OR+belief+OR+attitude+OR+enabler%2A+OR+Motivator%2A+OR+facilitator%2A+OR+encouragement+OR+predictor%2A+OR+determinant%2A+OR+engagement+OR+uptake+OR+initiation+OR+Use+OR+Utilisation+OR+Utilization+OR+Compliance+OR+Adherence&ac=no&sort=relevance) |
| **#2** | **"Pre-Exposure Prophylaxis"[Mesh] OR “pre-exposure prophylaxis" OR PrEP OR Truvada OR Descovy** | [23,805](https://pubmed.ncbi.nlm.nih.gov/?term=%22Pre-Exposure+Prophylaxis%22%5BMesh%5D+OR+%E2%80%9Cpre-exposure+prophylaxis%22+OR+PrEP+OR+Truvada+OR+Descovy&ac=no&sort=relevance) |
| **#3** | **“men who have sex with men” OR MSM OR Gay* OR “male couple*” OR homosexual* OR “transgender wom*” OR “trans wom*” OR “bisexual men”** | [64,629](https://pubmed.ncbi.nlm.nih.gov/?term=%E2%80%9Cmen+who+have+sex+with+men%E2%80%9D+OR+MSM+OR+Gay%2A+OR+%E2%80%9Cmale+couple%2A%E2%80%9D+OR+homosexual%2A+OR+%E2%80%9Ctransgender+wom%2A%E2%80%9D+OR+%E2%80%9Ctrans+wom%2A%E2%80%9D+OR+%E2%80%9Cbisexual+men%E2%80%9D&ac=no&sort=relevance) |
| **#4** | **Iowa OR “Midwest region” OR “Midwestern region” OR “Midwest state*” OR** [**Illinois**](https://www.bls.gov/regions/midwest/illinois.htm) **OR** [**Indiana**](https://www.bls.gov/regions/midwest/indiana.htm) **OR** [**Kansas**](https://www.bls.gov/regions/mountain-plains/kansas.htm) **OR** [**Michigan**](https://www.bls.gov/regions/midwest/michigan.htm) **OR Minnesota OR Missouri OR Nebraska OR “North Dakota” OR Ohio OR “South Dakota” OR Wisconsin** | [1,448,163](https://pubmed.ncbi.nlm.nih.gov/?term=Iowa+OR+%E2%80%9CMidwest+region%E2%80%9D+OR+%E2%80%9CMidwestern+region%E2%80%9D+OR+%E2%80%9CMidwest+state%2A%E2%80%9D+OR+Illinois+OR+Indiana+OR+Kansas+OR+Michigan+OR+Minnesota+OR+Missouri+OR+Nebraska+OR+%E2%80%9CNorth+Dakota%E2%80%9D+OR+Ohio+OR+%E2%80%9CSouth+Dakota%E2%80%9D+OR+Wisconsin&ac=no&sort=relevance) |
| **#5** | **(((Barrier* OR problem* OR reluctan* OR concern* OR stigma OR perception OR belief OR attitude OR enabler* OR Motivator* OR facilitator* OR encouragement OR predictor* OR determinant* OR engagement OR uptake OR initiation OR Use OR Utilisation OR Utilization OR Compliance OR Adherence) AND ("Pre-Exposure Prophylaxis"[Mesh] OR "pre-exposure prophylaxis" OR PrEP OR Truvada OR Descovy)) AND ("men who have sex with men" OR MSM OR Gay* OR "male couple*" OR homosexual* OR "transgender wom*" OR "trans wom*" OR "bisexual men")) AND (Iowa OR "Midwest region" OR "Midwestern region" OR "Midwest state*" OR Illinois OR Indiana OR Kansas OR Michigan OR Minnesota OR Missouri OR Nebraska OR "North Dakota" OR Ohio OR "South Dakota" OR Wisconsin)** | [340](https://pubmed.ncbi.nlm.nih.gov/?term=%28%28%28Barrier%2A+OR+problem%2A+OR+reluctan%2A+OR+concern%2A+OR+stigma+OR+perception+OR+belief+OR+attitude+OR+enabler%2A+OR+Motivator%2A+OR+facilitator%2A+OR+encouragement+OR+predictor%2A+OR+determinant%2A+OR+engagement+OR+uptake+OR+initiation+OR+Use+OR+Utilisation+OR+Utilization+OR+Compliance+OR+Adherence%29+AND+%28%22Pre-Exposure+Prophylaxis%22%5BMesh%5D+OR+%22pre-exposure+prophylaxis%22+OR+PrEP+OR+Truvada+OR+Descovy%29%29+AND+%28%22men+who+have+sex+with+men%22+OR+MSM+OR+Gay%2A+OR+%22male+couple%2A%22+OR+homosexual%2A+OR+%22transgender+wom%2A%22+OR+%22trans+wom%2A%22+OR+%22bisexual+men%22%29%29+AND+%28Iowa+OR+%22Midwest+region%22+OR+%22Midwestern+region%22+OR+%22Midwest+state%2A%22+OR+Illinois+OR+Indiana+OR+Kansas+OR+Michigan+OR+Minnesota+OR+Missouri+OR+Nebraska+OR+%22North+Dakota%22+OR+Ohio+OR+%22South+Dakota%22+OR+Wisconsin%29&ac=no&sort=relevance) |

**Table S1: Search strategy for CINAHL PLUS (EBSCO)**

| **SEARCH** | **ALL TERMS** | **HIT** |
| --- | --- | --- |
| **#1** | **Barrier* OR problem* OR reluctan* OR concern* OR stigma OR perception OR belief OR attitude OR enabler* OR Motivator* OR facilitator* OR encouragement OR predictor* OR determinant* OR engagement OR uptake OR initiation OR Use OR Utilisation OR Utilization OR Compliance OR Adherence** | 2,682,501 |
| **#2** | **“pre-exposure prophylaxis” OR PrEP OR Truvada OR Descovy** | 5,214 |
| **#3** | **“men who have sex with men” OR MSM OR Gay* OR “male couple*” OR homosexual* OR “transgender wom*” OR “trans wom*” OR “bisexual men”** | 35,252 |
| **#4** | **Iowa OR “Midwest region” OR “Midwestern region” OR “Midwest state*” OR** [**Illinois**](https://www.bls.gov/regions/midwest/illinois.htm) **OR** [**Indiana**](https://www.bls.gov/regions/midwest/indiana.htm) **OR** [**Kansas**](https://www.bls.gov/regions/mountain-plains/kansas.htm) **OR** [**Michigan**](https://www.bls.gov/regions/midwest/michigan.htm) **OR Minnesota OR Missouri OR Nebraska OR “North Dakota” OR Ohio OR “South Dakota” OR Wisconsin** | 112,540 |
| **#5** | S1 AND S2 AND S3 AND S4 | 42 |

**Table S2: Search strategy for SCOPUS**

| **SEARCH** | **ALL TERMS** | **HIT** |
| --- | --- | --- |
| 1 | TITLE-ABS-KEY ( barrier* OR problem* OR reluctan* OR concern* OR stigma OR perception OR belief OR attitude OR enabler* OR motivator* OR facilitator* OR encouragement OR predictor* OR determinant* OR engagement OR uptake OR initiation OR use OR utili?ation OR compliance OR adherence ) | [14,835,903](https://www-scopus-com.gcu.idm.oclc.org/search/history/results.uri?origin=searchhistory&shid=2) |
| 2 | TITLE-ABS-KEY ( "pre-exposure prophylaxis" OR prep OR truvada OR descovy ) | [16,826](https://www-scopus-com.gcu.idm.oclc.org/search/history/results.uri?origin=searchhistory&shid=2) |
| 3 | ITLE-ABS-KEY ( "men who have sex with men" OR msm OR gay* OR "male couple*" OR homosexual* OR "transgender wom*" OR "trans wom*" OR "bisexual men" ) | [104,660](https://www-scopus-com.gcu.idm.oclc.org/search/history/results.uri?origin=searchhistory&shid=3) |
| 4 | TITLE-ABS-KEY ( iowa OR "midwest region" OR "midwestern region" OR "midwest state*" OR illinois OR indiana OR kansas OR michigan OR minnesota OR missouri OR nebraska OR "north dakota" OR ohio OR "south dakota" OR wisconsin ) | 359,352 |
| 5 | (barrier* OR problem* OR reluctan* OR concern* OR stigma OR perception OR belief OR attitude OR enabler* OR motivator* OR facilitator* OR encouragement OR predictor* OR determinant* OR engagement OR uptake OR initiation OR use OR utili?ation OR compliance OR adherence) AND (iowa OR "midwest region" OR "midwestern region" OR "midwest state*" OR illinois OR indiana OR kansas OR michigan OR minnesota OR missouri OR nebraska OR "north dakota" OR ohio OR "south dakota" OR wisconsin) AND ("men who have sex with men" OR msm OR gay* OR "male couple*" OR homosexual* OR "transgender wom*" OR "trans wom*" OR "bisexual men") AND ("pre-exposure prophylaxis" OR prep OR truvada OR descovy) | [76](https://www-scopus-com.gcu.idm.oclc.org/search/history/results.uri?origin=searchhistory&shid=7) |

**Table S1: Search strategy for Web of Science**

| **SEARCH** | **ALL TERMS** | **HIT** |
| --- | --- | --- |
| **#1** | **ALL=(Barrier* OR problem* OR reluctan* OR concern* OR stigma OR perception OR belief OR attitude OR enabler* OR Motivator* OR facilitator* OR encouragement OR predictor* OR determinant* OR engagement OR uptake OR initiation OR Use OR Utilisation OR Utilization OR Compliance OR Adherence)** | [30,012,191](https://www.webofscience.com/wos/woscc/summary/772f020c-3234-44cd-a541-9aaa7c8ce571-7e117199/relevance/1) |
| **#2** | **ALL=(“pre-exposure prophylaxis” OR PrEP OR Truvada OR Descovy)** | [24,126](https://www.webofscience.com/wos/woscc/summary/008fe5d8-9832-468e-b0df-47df90d51769-7e1151c6/relevance/1) |
| **#3** | **ALL=(“men who have sex with men” OR MSM OR Gay* OR “male couple*” OR homosexual* OR “transgender wom*” OR “trans wom*” OR “bisexual men”)** | [203,535](https://www.webofscience.com/wos/woscc/summary/45c7b069-a3d0-4917-8fcc-44b197dd3a17-7e1163f2/relevance/1) |
| **#4** | **ALL=(Iowa OR “Midwest region” OR “Midwestern region” OR “Midwest state*” OR Illinois OR Indiana OR Kansas OR Michigan OR Minnesota OR Missouri OR Nebraska OR “North Dakota” OR Ohio OR “South Dakota” OR Wisconsin)** | [3,612,649](https://www.webofscience.com/wos/woscc/summary/2c7c9857-f5cf-42ff-a0b7-cba15c25e658-7e11679f/relevance/1) |
| **#5** | **#1 AND #2 AND #3 AND #4** | [289](https://www.webofscience.com/wos/woscc/summary/8e1c522e-f990-407f-abd1-ac6e4d2f8ae5-7e117bf1/relevance/1) |

**Table S1: Search strategy for PsycINFO (ProQuest)**

| **SEARCH** | **ALL TERMS** | **HIT** |
| --- | --- | --- |
| **#1** | Barrier* OR problem* OR reluctan* OR concern* OR stigma OR perception OR belief OR attitude OR enabler* OR Motivator* OR facilitator* OR encouragement OR predictor* OR determinant* OR engagement OR uptake OR initiation OR Use OR Utilisation OR Utilization OR Compliance OR Adherence | [**2,755,318**](https://www.proquest.com/recentsearches.recentsearchtabview.recentsearchesgridview.scrolledrecentsearchlist.checkdbssearchlink_0:rerunsearch/E6FCD92E4F9849F6PQ/None?site=psycinfo&t:ac=RecentSearches) |
| **#2** | [“pre-exposure prophylaxis” OR PrEP OR Truvada OR Descovy](https://www.proquest.com/recentsearches.recentsearchtabview.recentsearchesgridview.scrolledrecentsearchlist.checkdbssearchlink:rerunsearch/6A8EF69CA9304043PQ/None?site=psycinfo&t:ac=RecentSearches) | [**2,498**](https://www.proquest.com/recentsearches.recentsearchtabview.recentsearchesgridview.scrolledrecentsearchlist.checkdbssearchlink_0:rerunsearch/6A8EF69CA9304043PQ/None?site=psycinfo&t:ac=RecentSearches) |
| **#3** | [“men who have sex with men” OR MSM OR Gay* OR ("male couple" OR "male couples") OR homosexual* OR ("transgender woman" OR "transgender women") OR ("trans woman" OR "trans women") OR “bisexual men”](https://www.proquest.com/recentsearches.recentsearchtabview.recentsearchesgridview.scrolledrecentsearchlist.checkdbssearchlink:rerunsearch/F80777DA11124E56PQ/None?site=psycinfo&t:ac=RecentSearches) | [**57,941**](https://www.proquest.com/recentsearches.recentsearchtabview.recentsearchesgridview.scrolledrecentsearchlist.checkdbssearchlink_0:rerunsearch/F80777DA11124E56PQ/None?site=psycinfo&t:ac=RecentSearches) |
| **#4** | [Iowa OR “Midwest region” OR “Midwestern region” OR ("midwest states") OR Illinois OR Indiana OR Kansas OR Michigan OR Minnesota OR Missouri OR Nebraska OR “North Dakota” OR Ohio OR “South Dakota” OR Wisconsin](https://www.proquest.com/recentsearches.recentsearchtabview.recentsearchesgridview.scrolledrecentsearchlist.checkdbssearchlink:rerunsearch/26AE292D8F904AD1PQ/None?site=psycinfo&t:ac=RecentSearches) | [**496,143**](https://www.proquest.com/recentsearches.recentsearchtabview.recentsearchesgridview.scrolledrecentsearchlist.checkdbssearchlink_0:rerunsearch/26AE292D8F904AD1PQ/None?site=psycinfo&t:ac=RecentSearches) |
| **#5** | (Barrier* OR problem* OR reluctan* OR concern* OR stigma OR perception OR belief OR attitude OR enabler* OR Motivator* OR facilitator* OR encouragement OR predictor* OR determinant* OR engagement OR uptake OR initiation OR Use OR Utilisation OR Utilization OR Compliance OR Adherence) AND (“pre-exposure prophylaxis” OR PrEP OR Truvada OR Descovy) AND (“men who have sex with men” OR MSM OR Gay* OR ("male couple" OR "male couples") OR homosexual* OR ("transgender woman" OR "transgender women") OR ("trans woman" OR "trans women") OR “bisexual men”) AND (Iowa OR “Midwest region” OR “Midwestern region” OR ("midwest states") OR Illinois OR Indiana OR Kansas OR Michigan OR Minnesota OR Missouri OR Nebraska OR “North Dakota” OR Ohio OR “South Dakota” OR Wisconsin) | [**103**](https://www.proquest.com/recentsearches.recentsearchtabview.recentsearchesgridview.scrolledrecentsearchlist.checkdbssearchlink_0:rerunsearch/AF5789CEAC044D0FPQ/None?site=psycinfo&t:ac=RecentSearches) |
